# The capsular vein as a novel surgical landmark for safe access to the temporomandibular joint: a retrospective cohort study

**DOI:** 10.1186/s40902-025-00480-x

**Published:** 2025-09-23

**Authors:** Jeong-Kui Ku, Jae-Young Kim, Jong-Ki Huh

**Affiliations:** 1 Department of Oral and Maxillofacial Surgery, Seoul National University Bundang Hospital, Seongnam-Si, Republic of Korea; 2 Department of Oral and Maxillofacial Surgery, Yonsei University College of Dentistry, Gangnam Severance Hospital, Seoul, Republic of Korea

**Keywords:** Temporomandibular joint surgery, Facial nerve injury, Surgical approach, Anatomic landmark, Capsular vein

## Abstract

**Background:**

Temporomandibular joint (TMJ) surgery carries a risk of facial nerve injury and intraoperative bleeding, especially in patients with anatomical distortion due to ankylosis or inflammation. This study introduces a novel anatomic landmark—the capsular vein—and evaluates a preauricular approach utilizing this vein to improve surgical safety.

**Methods:**

A retrospective cohort study was performed on 104 patients (109 TMJs) who underwent TMJ surgery between 2014 and 2022. During the approach, a vein consistently found at the superior aspect of the glenoid fossa (designated the capsular vein) was identified and ligated without requiring formal facial nerve dissection. The primary outcomes were the incidence and duration of postoperative facial nerve weakness. Secondary outcomes included the presence of any facial paresthesia and other postoperative symptoms.

**Results:**

The capsular vein was identified and ligated in all cases. No intraoperative bleeding requiring hemostasis (e.g., electrocautery) was observed. Temporary facial nerve weakness occurred in 3.8% of patients (*n* = 4). Additional complications included transient facial paresthesia (1.9%, *n* = 2) and headache (4.8%, *n* = 5), with no permanent deficits reported.

**Conclusion:**

The capsular vein serves as a reliable anatomic landmark for TMJ surgery, enabling a safe and efficient approach without the need for facial nerve dissection. Its use minimizes intraoperative bleeding and nerve injury, particularly in patients with ankylosis or severe inflammation, and may improve surgical outcomes across a variety of TMJ procedures.

## Introduction

Complication rates in temporomandibular joint (TMJ) surgery can vary widely among surgeons. Facial nerve injury is a well-recognized risk of TMJ surgery regardless of the surgical approach, due to normal anatomical variations in the course of the facial nerve branches or inadvertent traction from aggressive retraction [1, 2]. To minimize the risk of facial nerve injury, several surgical approaches to the TMJ have been described, including preauricular, endaural, retromandibular, and intraoral approaches. In 2016, a “vascular-guided multilayer” preauricular approach was demonstrated to avoid facial nerve injury [3]. The preauricular approach is one of the most commonly preferred methods, and the addition of a “hockey-stick” skin incision modification can improve anterior and lateral exposure [4].

Severe inflammatory conditions of the TMJ can cause degenerative changes in the joint tissues, with an increased inflammatory infiltrate and accompanying angiogenesis. During such inflammatory reactions, immune cells secrete pro-angiogenic factors that promote neovascularization [5]. In addition, chronic inflammation with destruction of the synovial lining and bone is a common cause of fibrous or bony ankylosis of the TMJ. The incidence of facial nerve injury increased significantly in the bony ankylosis patients during the TMJ surgery [6], as well as the fibro-osseous ankylosis [7]. Another important consideration in TMJ ankylosis surgery is designing the ostectomy such that it restores the optimal relationship between the articular fossa and the condylar head. This relationship is also a key factor when positioning a TMJ prosthesis to replace the articular fossa and condylar head [8]. However, no specific anatomical landmark has yet been proposed to guide surgeons with respect to the shape of the articular fossa or the position of the condylar head before making the capsular incision.

The aim of this study was to introduce a novel anatomic landmark, termed the “capsular vein,” which lies at the uppermost point of the articular fossa just superficial to the TMJ capsule. We also describe a modified preauricular surgical approach that avoids cauterization of any vessels except the capsular vein. The clinical outcomes of this technique are reported, demonstrating that by ligating only the capsular vein, the risk of nerve damage and intraoperative bleeding can be minimized across various TMJ surgeries.

## Material and methods

### Study design and sample

This retrospective study was approved by the institutional review board of Yonsei University Gangnam Severance Hospital (IRB No. 3-2022-0123) and was conducted according to the principles of the Declaration of Helsinki.

This retrospective cohort study was conducted on consecutive patients who received the TMJ surgery in the Department of Oral and Maxillofacial Surgery, Gangnam Severance Hospital, Korea, from January 2014 to March 2022. The inclusion criteria were as follows: (1) patients aged over 19 years, (2) absence of uncontrolled systemic disease, and (3) a minimum postoperative follow-up period of 6 months. The exclusion criteria were as follows: (1) emergency surgery due to TMJ fracture, (2) a history of previous open TMJ surgery, and (3) prior arthroscopic procedures. All operations were performed by a single experienced surgeon (with over 20 years of experience) following a standardized surgical protocol. Clinical information was obtained through retrospective chart review, including any subjective postoperative symptoms related to the auriculotemporal or facial nerves.

During the surgical approach, special attention was given to identifying a distinct vein at the superior aspect of the glenoid fossa. Intraoperative assessment confirmed the consistent presence of a single vein at this location in all cases; this vessel was designated as the “capsular vein.” No anatomical variations such as absence or duplication of the vein were observed. The reproducible vein was isolated and ligated without the need for direct dissection or visualization of the facial nerve, thereby serving as a reliable anatomic landmark to facilitate a safe and efficient approach.

The primary outcomes evaluated were the incidence and duration of postoperative facial nerve weakness. Secondary outcomes included the presence of any facial paresthesia and other subjective symptoms in the operative region.

### Representative surgical procedure with reference to the capsular vein

**Fig. 1:**
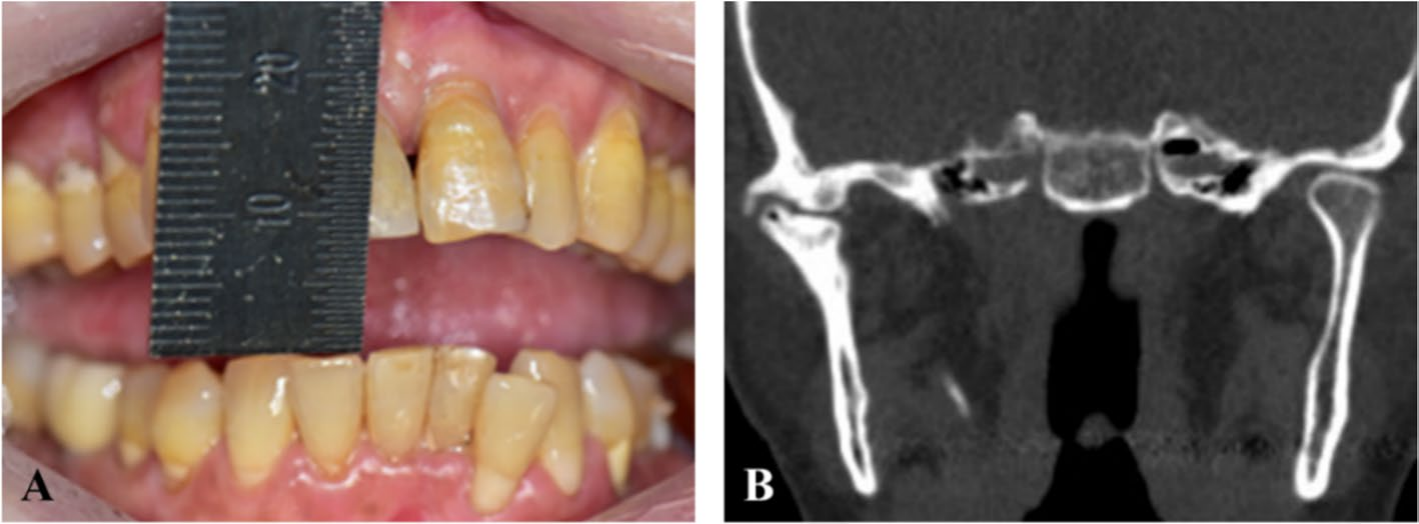
Preoperative images of ankylosis patient due to a facial trauma 2 years ago. **A** Maximum mouth opening was less than 9 mm. **B** Due to fibrous ankylosis and heterotopic bone formation of right TMJ, both condylar head and glenoid fossa were significantly deformed compared with the contralateral side

A 63-year-old female suffered the limitation of opening (< 9 mm) after facial trauma 2 years ago (Fig. 1). Cone-beam computed tomography (CBCT) showed a significantly deformed right TMJ; the patient was diagnosed with TMJ ankylosis and scheduled for arthroplasty of the right joint.

**Fig. 2:**
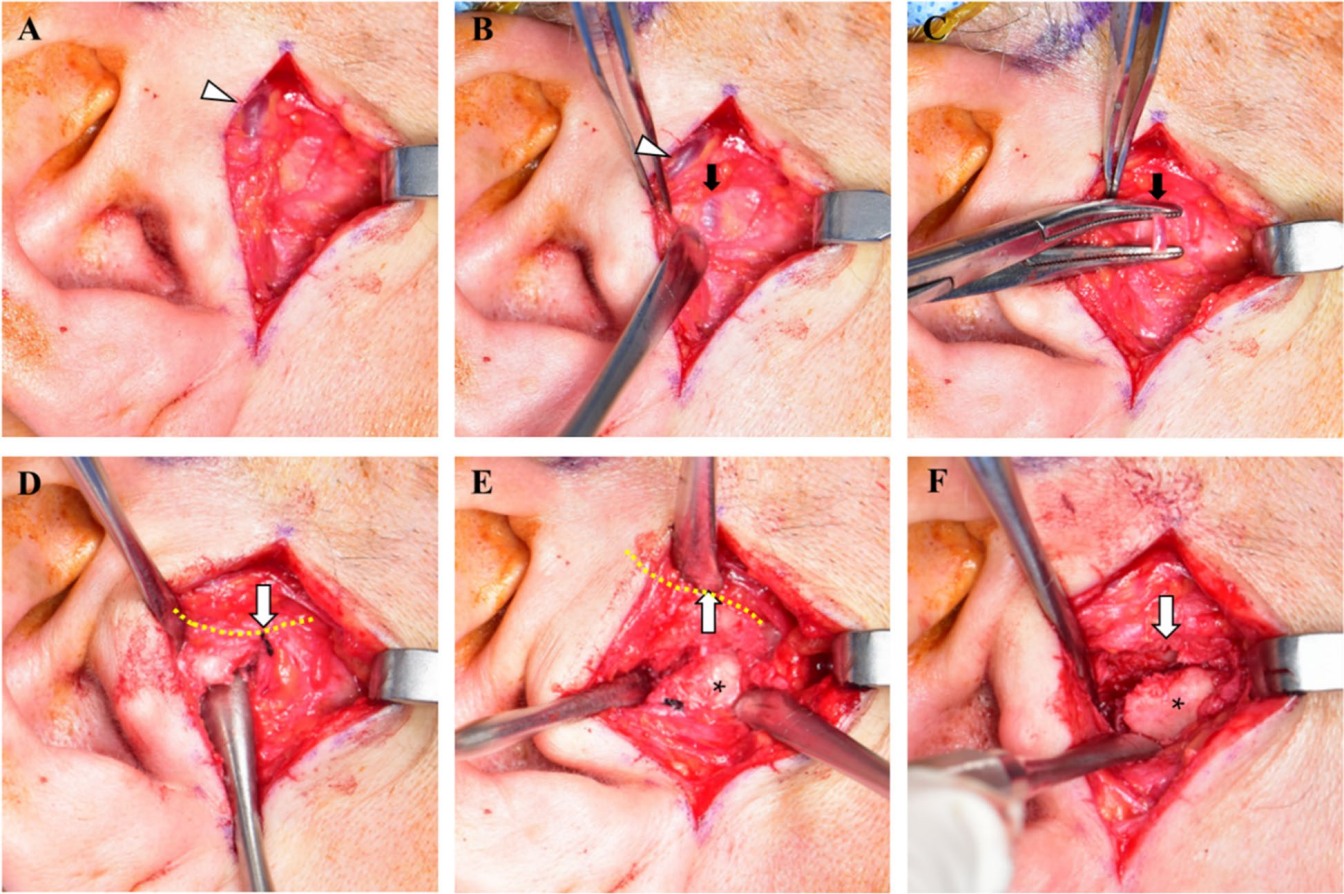
Intraoperative images of preauricular approach for TMJ ankylosis by using the capsular vein as a special reference. **A** After dissection under the skin flap through preauricular incision, the superficial temporal vein (arrow head) was exposed on the temporoparietal fascia (superficial temporalis fascia) through the skin and subcutaneous tissue. **B** The capsular vein (black arrow) was detected with careful dissection into the temporoparietal fascia. **C** The vein (black arrow) was isolated and ligated. This vein was the only sacrificed vessel through this approach. **D** The frontal and zygomatic branches of facial nerve were detected by a nerve stimulator in the anterior part (yellow dotted line) at this level of layer. Based on the location of the ligated vein (arrow), the posterior superior joint space could be approached. **E** The facial nerve was anteriorly retracted beyond the yellow dotted line, and the ligated vein was superiorly retracted (arrow). The lateral pole of the condylar head was exposed (asterisk). **F** The condylar head (asterisk) could be separated from the articular eminence anteriorly based on the location of the capsular vein (arrow) as the midpoint of the glenoid fossa

According to a standard preauricular approach, a skin incision was made along the crease between the mid-portion of the earlobe and the level of the helix. This incision was then extended superiorly and obliquely for approximately 1 cm at an angle of about 120°. Dissection was carried out using mosquito forceps through the subcutaneous tissue in an avascular plane (the superficial musculoaponeurotic system, SMAS). After reflecting the skin and subcutaneous flap, blunt dissection was continued along the superficial temporal fascia in the same plane. During this dissection, the superficial temporal vein was identified and retracted away from the surgical field (Fig. 2A).

After identifying the temporalis fascia at the midpoint of the incision via blunt dissection, the deep temporal fascia was then located by tactile feedback on the periosteum of the zygomatic arch. Within this dense layer, careful blunt dissection was carried out in a downward direction to find a small vein running vertically within the layer (Fig. 2B). The vein was isolated and ligated with 3–0 silk (Fig. 2C). Use of a nerve stimulator to locate the facial nerve was not necessary at this stage, because the nerve lies in the same fascial layer as the capsular vein (Fig. 2D). After confirming with a nerve stimulator that the facial nerve was not present in the immediate area, the temporalis fascia (containing the ligated capsular vein) was incised and elevated to expose the TMJ capsule and the articular eminence (Fig. 2E).

Because the capsular vein was consistently located at the midpoint of the glenoid fossa (the most superolateral aspect of the fossa) in all cases, the site of ligation provided a useful reference for planning the capsular incision or bony ostectomy. This was particularly helpful in cases of ankylosis and in positioning components for total joint replacement, as the vein’s location roughly corresponds to the center of the condylar head in an intact joint (Fig. 2F).

## Results

A total of 104 patients (109 joints; 19 males and 85 females; mean age 41.2 ± 15.0 years) underwent TMJ surgeries during the study period. These included arthroplasty in 72 joints (66.1%), total joint replacement in 16 joints (14.7%), condylectomy in 9 joints (8.3%), and excision of synovial chondromatosis or giant cell tumor in 11 joints (10.1%) (Table 1). The capsular vein was identified and ligated in all patients. During the approach to the TMJ capsule, no bleeding was encountered that required electrocautery or other hemostatic measures.

**Table 1:** Summary of diagnosis and surgeries for TMJ

Diagnosis	*N* (%, joints)	TMJ surgery	*N* (%, joint)
Osteoarthritis and degenerative joint disease	55 (50.5%)	Arthroplasty	72 (66.1%)
Bony/fibrous ankylosis	25 (22.9%)		
Synovial chondromatosis	15 (13.8%)	Total joint replacement	16 (14.7%)
Osteochondroma	8 (7.3%)		
Myositis ossificans	2 (1.8%)	Condylectomy	9 (8.3%)
Condylar hyperplasia	1 (0.9%)		
Giant cell tumor	1 (0.9%)	Mass excision	11 (10.1%)
Hemifacial hypertrophy	1 (0.9%)		

Postoperative complications occurred in 11 patients (10.1%) (Table 2). Four patients (3.8%) developed temporary weakness of the temporal branch of the facial nerve; all cases of facial nerve weakness resolved spontaneously within 2 weeks to 9 months. Two patients (1.9%) experienced numbness in the preauricular region, lasting for 4.5 months and 7 months, respectively. Five patients (4.8%) reported headaches, which resolved within 2 weeks to 2 months. No patient developed any permanent complication following TMJ surgery.

**Table 2:** Postoperative complications and duration

Complications	Sex/age	Duration	Surgery
Facial nerve weakness (*n* = 4)	F/34	2 weeks	Mass excision for osteochondroma
	F/30	2 months	Arthroplasty for osteoarthritis
	F/34	5 months	Total joint replacement for ankylosis
	M/42	9 months	Total joint replacement for myositis ossificans
Numbness (*n* = 2)	F/22	4.5 months	Arthroplasty for osteoarthritis
	F/61	7 months	Arthroplasty for ankylosis
Headache (*n* = 5)	F/33	2 weeks	Total joint replacement for ankylosis
	M/63 F/27	1 month	Arthroplasty for osteoarthritis for 2 patients Arthroplasty for degenerative joint disease for 1 patient
	F/36	1.5 month	Arthroplasty for synovial chondromatosis and osteoarthritis

## Discussion

Over the past two decades of TMJ surgeries, we have consistently observed a vein passing over the TMJ capsule. The capsular vein described in this study has not been clearly defined in the literature; however, it is presumed to be a branch of the middle temporal vein. In all patients, we observed that the capsular vein runs vertically across the middle of the articular fossa in the same fascial plane as the frontal branch of the facial nerve, making it a useful intraoperative guide to avoid nerve injury. In our series, identifying and ligating this vessel during the TMJ approach was associated with no significant intraoperative bleeding and no permanent facial nerve weakness, and it reduced the need for extensive dissection to locate the facial nerve. These findings suggest that using the capsular vein as a landmark allows the TMJ to be approached more efficiently and safely.

Maxillofacial surgeons frequently operate on a wide variety of TMJ pathologies. Although many skin incisions have been suggested, the fundamental deep approach is similar because the key structures lie within the same anatomical layers [9]. The preauricular approach remains one of the preferred options [4, 10]. The hockey-stick modification can improve the anterior and lateral exposure and results in safe and temporary impairment of function [6]. Despite the evolution of numerous approaches to the TMJ, the frontal (temporal) branch of the facial nerve remains at risk for injury during dissection. Opinions differ regarding the exact course and safe region of this nerve branch, and considerable variation exists in anatomical descriptions [9]. During TMJ surgery, it is essential to identify and avoid the facial nerve branches to prevent injury; however, facial nerve injury following to the maxillofacial region ranges from 0 to 48% [1]. The highly variable course of the temporal branch makes it particularly susceptible to injury during TMJ surgery [11]. The risk of facial nerve injury is especially elevated in patients with TMJ ankylosis [6, 7]. In severe inflammatory conditions like ankylosis, the normal fascial planes can be distorted, making it difficult to maintain the correct dissection plane. This significantly increases the risk of nerve injury, even though surgeons are aware that the temporal branch typically lies 8–35 mm anterior to the external auditory meatus, deep to the temporoparietal fascia at the level of the zygomatic arch [12]. Using our described technique, after the capsular vein was ligated, the temporal branch of the facial nerve could be consistently identified with a nerve stimulator on the anterior or anteromedial side of the ligated vein in all patients. We emphasize that this procedure obviates the need for a separate dissection to find the facial nerve, thereby reducing the risk of direct nerve exposure or injury. Nevertheless, surgeons should still take care to avoid excessive retraction or stretching of the tissues, as this can cause neurapraxia of the nerve.

Arthroplasty—which was the most commonly performed procedure (66.1% of cases) in this study—is among the oldest techniques for TMJ surgery, having originated in the nineteenth century. The early form of this procedure involved a simple resection to separate the mandibular ramus from the skull base. Because that approach often resulted in a high rate of re-ankylosis, later modifications increased the gap between the resected bone segments (to at least 10 mm), leading to the development of the gap arthroplasty (GA) technique [13]. To create such a large gap, the surgical resection typically includes not only the condyle but also a portion of the glenoid fossa. Because the glenoid fossa is a deep concavity in the temporal bone, preserving or re-establishing its shape may be advantageous for TMJ function. Wang et al. reported in an in vivo study that the presence and proximity of the developing mandibular condyle are essential for normal development of the glenoid fossa [14]. In cases with normal anatomy, we found that the ligated capsular vein was typically positioned at the midpoint of the glenoid fossa, corresponding roughly to the center of the condylar head. Therefore, the capsular vein can serve as a useful intraoperative reference point during arthroplasty. Importantly, the capsular vein courses vertically across the lateral capsule at the most superior point of the glenoid fossa. This consistent anatomical location allows it to serve as a valuable reference even in cases where normal anatomy is disrupted, such as bony ankylosis, severe inflammation, loss of the condylar head, or displaced condylar fractures. Furthermore, as long as the condyle and capsule are present (e.g., in conditions other than complete bony ankylosis), this vein could be used as a landmark for the capsular incision to access the superior joint space—for example, to help locate and remove pathologic nodules in synovial chondromatosis.

This study has several limitations. First, it was a retrospective analysis without a control group, which inherently introduces potential selection bias and limits the strength of comparative conclusions. All patients in our series underwent the same surgical technique, so we could not directly compare outcomes between the capsular vein-guided approach and other conventional approaches that involve facial nerve dissection or different landmarks. Second, the capsular vein has not been clearly defined in anatomical literature, and we did not perform cadaveric dissection or dedicated imaging studies to validate its anatomy. Thus, its exact origin and course, as well as its consistent relationship to the facial nerve, remain to be confirmed. Looking ahead, further studies are needed to validate and expand upon our findings. Anatomical verification of the “capsular vein” through cadaveric dissection would help confirm its consistent presence, vascular origin, and precise anatomical relationship to the facial nerve and surrounding structures.

Despite these limitations, the capsular vein was consistently present and identifiable in all patients in our cohort. By using the capsular vein as an anatomic landmark to guide the surgical approach, we were able to minimize the risk of nerve damage and intraoperative bleeding, since this was the only vessel that required ligation. This technique also improved visualization of the TMJ capsule and articular eminence, allowing the surgeon to plan the capsular incision or ostectomy for procedures such as ankylosis release, total joint replacement, or other interventions based on the patient’s needs. Ultimately, our clinical results provide a foundation for future research on this structure. Multicenter studies with larger patient samples, as well as dedicated cadaveric dissection and high-resolution imaging to validate the vein’s anatomical characteristics, can build on our work to determine the broader applicability and surgical relevance of the capsular vein as a landmark for safe TMJ surgery.

## Conclusion

The capsular vein can be a useful guide for designing capsular incision and ostectomy for ankylosis and to avoid facial nerve injury during various TMJ surgeries. The technique of using the capsular vein is especially effective in patients with severe anatomical changes like fibrous/bony ankylosis or severe inflammation of the TMJ, as these conditions can increase the risk of nerve injury and severe anatomical variation. By minimizing the possibility of nerve damage and intraoperative bleeding, this anatomic reference provides an optimized surgical technique and improves patient outcomes.

## Data Availability

The data and materials are available upon request.
